# Association of Arterial Stiffness With Mid- to Long-Term Home Blood Pressure Variability in the Electronic Framingham Heart Study: Cohort Study

**DOI:** 10.2196/54801

**Published:** 2024-04-08

**Authors:** Xuzhi Wang, Yuankai Zhang, Chathurangi H Pathiravasan, Nene C Ukonu, Jian Rong, Emelia J Benjamin, David D McManus, Martin G Larson, Ramachandran S Vasan, Naomi M Hamburg, Joanne M Murabito, Chunyu Liu, Gary F Mitchell

**Affiliations:** 1 Department of Biostatistics Boston University School of Public Health Boston, MA United States; 2 Boston University's and National Heart, Lung, and Blood Institute's Framingham Heart Study Boston, MA United States; 3 Section of Cardiovascular Medicine, Department of Medicine Boston University Chobanian and Avedisian School of Medicine Boston, MA United States; 4 Department of Epidemiology Boston University School of Public Health Boston, MA United States; 5 Cardiology Division, Department of Medicine University of Massachusetts Chan Medical School Worcester, MA United States; 6 Department of Quantitative Health Sciences University of Massachusetts Chan Medical School Worcester, MA United States; 7 Section of Preventive Medicine and Epidemiology, Department of Medicine Boston University Chobanian and Avedisian School of Medicine Boston, MA United States; 8 Section of Vascular Biology Boston University Chobanian and Avedisian School of Medicine Boston, MA United States; 9 Section of General Internal Medicine, Department of Medicine Boston University Chobanian and Avedisian School of Medicine Boston, MA United States; 10 Cardiovascular Engineering Boston, MA United States

**Keywords:** arterial stiffness, mobile health, mHealth, blood pressure, blood pressure variability, risk factors

## Abstract

**Background:**

Short-term blood pressure variability (BPV) is associated with arterial stiffness in patients with hypertension. Few studies have examined associations between arterial stiffness and digital home BPV over a mid- to long-term time span, irrespective of underlying hypertension.

**Objective:**

This study aims to investigate if arterial stiffness traits were associated with subsequent mid- to long-term home BPV in the electronic Framingham Heart Study (eFHS). We hypothesized that higher arterial stiffness was associated with higher home BPV over up to 1-year follow-up.

**Methods:**

At a Framingham Heart Study research examination (2016-2019), participants underwent arterial tonometry to acquire measures of arterial stiffness (carotid-femoral pulse wave velocity [CFPWV]; forward pressure wave amplitude [FWA]) and wave reflection (reflection coefficient [RC]). Participants who agreed to enroll in eFHS were provided with a digital blood pressure (BP) cuff to measure home BP weekly over up to 1-year follow-up. Participants with less than 3 weeks of BP readings were excluded. Linear regression models were used to examine associations of arterial measures with average real variability (ARV) of week-to-week home systolic (SBP) and diastolic (DBP) BP adjusting for important covariates. We obtained ARV as an average of the absolute differences of consecutive home BP measurements. ARV considers not only the dispersion of the BP readings around the mean but also the order of BP readings. In addition, ARV is more sensitive to measurement-to-measurement BPV compared with traditional BPV measures.

**Results:**

Among 857 eFHS participants (mean age 54, SD 9 years; 508/857, 59% women; mean SBP/DBP 119/76 mm Hg; 405/857, 47% hypertension), 1 SD increment in FWA was associated with 0.16 (95% CI 0.09-0.23) SD increments in ARV of home SBP and 0.08 (95% CI 0.01-0.15) SD increments in ARV of home DBP; 1 SD increment in RC was associated with 0.14 (95% CI 0.07-0.22) SD increments in ARV of home SBP and 0.11 (95% CI 0.04-0.19) SD increments in ARV of home DBP. After adjusting for important covariates, there was no significant association between CFPWV and ARV of home SBP, and similarly, no significant association existed between CFPWV and ARV of home DBP (*P*>.05).

**Conclusions:**

In eFHS, higher FWA and RC were associated with higher mid- to long-term ARV of week-to-week home SBP and DBP over 1-year follow-up in individuals across the BP spectrum. Our findings suggest that higher aortic stiffness and wave reflection are associated with higher week-to-week variation of BP in a home-based setting over a mid- to long-term time span.

## Introduction

Nearly half of US adults have hypertension [1]. The 2017 American College of Cardiology/American Heart Association blood pressure (BP) guidelines recommended out-of-office self-monitoring with home BP measurements to assist with hypertension diagnosis and management. Moreover, home-based BP measurements are stronger predictors of cardiovascular risk than office-based measurements [2]. BP fluctuates in response to everyday activities including exercise, mental stress, sleep, and other environmental stimuli. Parati et al [3] defined several types of blood pressure variability (BPV), including short-term BPV (eg, ambulatory BP monitoring within 24 hours), midterm BPV (eg, day-to-day BP monitoring in at least 3 days), and long-term BPV (eg, visit-to-visit BP monitoring over weeks to years). Elevated short-term BPV from ambulatory BP monitoring is associated with a higher risk of cardiovascular outcomes and all-cause mortality [4]. Day-to-day home BPV over 1 [5,6] to 4 weeks [7,8] is associated with cardiovascular risk and may identify persons at risk for cognitive decline [9]. Long-term BPV is associated with adverse cardiovascular events and mortality even after accounting for mean BP [10,11] in persons with and without hypertension [12]. However, week-to-week home BPV that is measured over the course of up to 1 year has not been well defined in the literature. In our study, we define mid- to long-term home BPV as the week-to-week home BPV collected during up to 1-year follow-up. Limited data are available on mid- to long-term home BPV in association with cardiovascular risk.

Arterial stiffness may be 1 biological mechanism linking BPV to cardiovascular disease risk [13]. Higher arterial stiffness is associated with a higher risk for incident hypertension [14] and is associated with both short-term and long-term adverse health outcomes including coronary disease events and heart failure [15,16]. Increased long-term visit-to-visit systolic BPV may contribute to the progression of arterial stiffness, regardless of mean BP levels [17]. Short-term (24-hour) BPV is associated with arterial stiffness [18]. Studies of mid- to long-term home BPV and arterial stiffness are limited; in one study, home BPV was correlated with a measure of arterial wave reflection in persons with high normal BP or hypertension [19]. Evaluation of the association of direct measures of arterial stiffness and wave reflection with home BPV over a mid- to long-term span is needed [3].

BPV indices have been proposed to calculate the overall variability as well as specific BP patterns [3], including SD, coefficient of variation (CV), average real variability (ARV), and variability independent of the mean. In our study, we obtained the ARV of week-to-week home BP from participants in the electronic Framingham Heart Study (eFHS) who returned digital BP data over up to 1-year follow-up. We investigated the association between arterial stiffness traits and the mid- to long-term home BPV, that is, the ARV of week-to-week home systolic blood pressure (SBP) and diastolic blood pressure (DBP) over up to 1-year follow-up from the eFHS participants. We hypothesize that higher arterial stiffness is associated with higher mid- to long-term home BPV over up to 1-year follow-up.

## Methods

### Study Participants

The Framingham Heart Study (FHS) is a multigenerational cohort study that began in 1948 with the original cohort enrolling 5209 residents from Framingham, Massachusetts. In 1971, the offspring cohort enrolled 5214 participants who were offspring of the original cohort and the spouses of these offspring. The FHS enrolled the Third Generation (Gen 3) cohort (N=4095), which included the grandchildren of original cohort from 2002 to 2005. During the same time, the FHS recruited and enrolled the Omni 2 cohort comprised of 410 multiethnic participants, and the New Offspring Spouse cohort (n=103) comprised of previously unenrolled parents of the Gen 3 participants. The participants in the Gen 3, Omni 2, and New Offspring Spouse cohorts underwent research exams approximately every 6 to 8 years. At examination 3 (2016 to 2019) participants underwent arterial tonometry testing and participants who owned a smartphone (including iPhone 4S or higher with iOS version 8.2 or higher or an Android phone beginning October 30, 2017) were invited to enroll in the eFHS. The eFHS participants downloaded a smartphone app and for participants using an iPhone, a Nokia Withings digital BP cuff was provided for home BP monitoring (Multimedia Appendix 1). The Nokia Withings digital cuff has been cleared for marketing by the Food and Drug Administration and it has been validated [20,21]. The eFHS participants downloaded the eFHS smartphone app from the Apple Store with in-person help from the eFHS–trained staff or with written instructions provided by eFHS staff.

Participants with arterial tonometry measures who returned valid digital home BP readings as part of eFHS were eligible for inclusion in the study sample. A total of 3451 participants underwent arterial tonometry at examination 3. Of the 3451 participants, 2125 participated in the eFHS study. Among the eFHS participants, 1156 participants provided BP data using the digital BP cuff and the study smartphone app. We further excluded 299 participants for the following reasons: participants did not return BP measurements within the first 12 months of attending examination 3 (n=126), participants returned BP readings for <3 weeks (n=153), or participants had missing values in tonometry measures or covariates (n=20). After exclusion, 857 participants remained for subsequent statistical analysis (Multimedia Appendix 2). We further compared our final study sample with those who did not enroll in eFHS and those ineligible for inclusion in our final sample.

### Assessment of Arterial Tonometry Measures

We examined 3 measures of arterial stiffness and wave reflection obtained from arterial tonometry including carotid-femoral pulse wave velocity (CFPWV), central forward pressure wave amplitude (FWA), and reflection coefficient (RC). Trained sonographers obtained the tonometry measurements using a standard protocol previously reported [22,23]. We chose to investigate CFPWV as this measure is the standard noninvasive measure of arterial stiffness recommended for vascular research [24]. CFPWV was calculated from carotid-femoral transit time delay and carotid-femoral transit distance adjusted for parallel transmission in the brachiocephalic and carotid arteries and aortic arch [23]. Wave separation was performed in the time domain [23]. FWA was defined as the amplitude of the forward pressure wave. RC was defined as the ratio of backward and forward wave amplitudes. As compared with CFPWV, FWA is comparably sensitive to aortic wall stiffness but is more sensitive to aortic diameter [14]. RC was assessed as a measure of relative wave reflection as described previously [25]. The magnitude of the RC relies on the degree of impedance mismatch between proximal and distal vessels. Impedance matching reduces RC and hence the amount of wave reflection at the interface between the aorta and branch vessels, which may result in the transmission of excessive pulsatile energy into the microcirculation where it can cause damage [25].

### Assessment of Week-to-Week Home BP Measurement and BP Variability

eFHS participants were asked to measure and transmit home BP readings once each week using the Withings-Nokia digital BP cuff for up to 1 year following enrollment. Multimedia Appendix 1 displays the timeline of data collection for arterial tonometry measures, as well as for weekly home BP readings. eFHS staff demonstrated proper use of the digital BP cuff while the participant was in the Research Center. Written instructions were also provided as some participants chose to set up the digital cuff at home. Participants were asked to take a BP reading at about the same time of day and the same day of the week each week. Participants were advised to sit in a comfortable position and rest for 5 minutes before each BP reading was measured. Participants were instructed to avoid taking BP readings after doing exercise or consuming caffeinated beverages. All BP recordings were date- and time-stamped. We conducted quality control procedures in the following way: BP readings taken at the research center during training by eFHS staff, duplicate observations with identical BP readings, observations with values that likely represented spurious results including observations with DBP>SBP, SBP>250, DBP>140, SBP<70, or DBP<40 were excluded. To reduce the bias of week-to-week home BPV, we included eFHS participants who had BP readings for at least 3 weeks [26]. Because we aimed to investigate the association of antecedent arterial stiffness with week-to-week home BPV over a mid- to long-term time span, we excluded BP measurements assessed more than 1 year after enrollment.

Several common variability measures have been used to assess BPV, including SD and CV. Compared with these BPV indices, ARV not only considers the dispersion of the BP time series around the mean but also accounts for the order of BP readings [27,28]. Furthermore, SD and CV are sensitive to long-range variation, such as a progressive increase or decrease in BP, while being less sensitive to measurement-to-measurement variability. ARV was first proposed to calculate the 24-hour ambulatory BPV. In our study, we proposed to apply the ARV to week-to-week home SBP and DBP measurements over a mid- to long-term time span defined as up to 1 year. ARV was calculated as the average of absolute differences between the adjacent BP readings using the following formula:



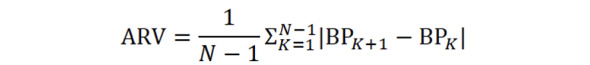



where *N* is the number of weekly BP measurements and *K* is the order of weekly BP measurements.

### Covariates

Clinical and laboratory variables were collected during examination 3 at the research center. BMI was calculated by dividing body weight (kg) by height (meters) squared. The current smoking variable was defined as self-reported smoking 1 or more cigarettes per day on average in the year before the examination. Lipid-lowering treatment variable was defined as a self-report of receiving lipid treatment in the past month before the examination. Antihypertensive medication variable was defined as a self-report of taking antihypertensive medication in the past month before the examination. Diabetes was defined as fasting plasma glucose ≥126 mg/dL or self-reported use of medications for diabetes. SBP and DBP were measured by averaging 2 readings while the participant was seated in a chair following a minimum of 5 minutes of rest at the research center. Hypertension was defined as SBP ≥130 mm Hg, DBP ≥80 mm Hg, or self-reported use of antihypertensive medications. Pulse pressure (PP) was calculated by taking the difference between SBP and DBP (SBP-DBP). Mean arterial pressure (MAP) was calculated as the integrated mean of the calibrated brachial pressure waveform at the time of the arterial tonometry test at examination 3. Fasting total cholesterol, high-density lipoprotein cholesterol, triglycerides, and blood glucose were also obtained during examination 3 at the research center.

### Statistical Analyses

We reported mean and SD for continuous variables with approximately normal distributions, median and IQR for continuous variables with skewed distributions, and frequency and proportion for categorical variables. We conducted linear regressions to investigate the relations between arterial stiffness traits (predictors) and home BPV as estimated by ARV (outcomes). Prior to regression analysis, CFPWV was inverse-transformed to reduce heteroscedasticity and skewness and was then multiplied by –1000 to convert the units to milliseconds per meter and restore directionality. Therefore, the transformed CFPWV was expressed as –1000/CFPWV. To facilitate comparison and interpretation, all predictors and outcomes were scaled to unit SD in the regression analysis.

For each of the arterial stiffness traits (CFPWV, FWA, and RC), the association with home BPV was evaluated using separate linear models for ARV indices derived from home SBP and home DBP. In the base models, we performed linear regressions adjusting for age, age-squared, and sex. We adjusted for age-squared because previous studies observed that many stiffness measures and BP measures showed nonlinear age relations [22]. In the multivariable models, all base models were further adjusted for the following covariates: BMI, height, heart rate, total cholesterol, high-density lipoprotein cholesterol, triglycerides, lipid-lowering treatment, fasting glucose, diabetes, current smoking, and antihypertensive medication. The multivariable model helps us determine if there is a residual association of stiffness after adjusting for the other partially downstream and partially independent or upstream effects. In addition, in secondary analysis, to account for any pressure dependence of stiffness variables, we investigated how MAP influenced the associations between arterial stiffness traits and ARV of home BP by further adjusting for MAP in the multivariable model.

All statistical analyses were conducted using R (version 4.0; R Foundation for Statistical Computing). We used 2-tailed *P*<.05 for significance.

### Ethical Considerations

All study participants provided informed consent. The eFHS and FHS protocols were approved by the institutional review board at the Boston University Medical Center (H-36586 and H-32132). We confirm that we have the permission to use the data.

## Results

### Participant Characteristics

Characteristics of the study sample are summarized in Table 1. Our study sample consisted of 857 participants who were middle-aged on average, with a moderate prevalence of hypertension. Average SBP and DBP were within the normal range, whereas approximately 1 in 5 participants reported taking antihypertensive medications. Participants were overweight on average, whereas the prevalence of smoking and diabetes was low. Compared with the BP readings at examination 3, the average home SBP during eFHS over 1-year of follow-up was slightly higher, and the average home DBP was similar. Compared with the FHS attendees who did not enroll in eFHS and the eFHS participants ineligible for our final analysis, the eFHS participants in our study sample were generally healthier, more likely to be female, and had a lower prevalence of risk factors. In addition, the final study sample had a lower mean CFPWV, larger mean RC, and lower mean SBP compared with the FHS participants who did not enroll in eFHS. However, compared with the ineligible eFHS participants, while our final study sample also had a larger mean RC, there were no significant differences in terms of CFPWV and SBP (Multimedia Appendix 3).

**Table 1:** Characteristics of the study sample (N=857).

Characteristics or covariates			Variables
**At the time of research examination 3**			
	Age (years), mean (SD)		54 (9)
	Sex (female), n (%)		508 (59)
	BMI (kg/m^2^), mean (SD)		27.6 (4.79)
	**Race and ethnicity, n (%)**		
		Asian	17 (2)
		Black	18 (2.1)
		Hispanic	19 (2.2)
		White	786 (91.7)
		Other	17 (2)
	Height (inches), mean (SD)		66.6 (3.52)
	Total cholesterol (mg/dL), mean (SD)		190 (35.8)
	High-density lipoprotein cholesterol (mg/dL), mean (SD)		62.0 (19.9)
	Triglycerides (mg/dL), median (IQR)		87 (65-104)
	Fasting blood glucose (mg/dL), mean (SD)		97.9 (17.9)
	Heart rate (bpm), mean (SD)		58 (9.08)
	Antihypertensive use, n (%)		189 (22.1)
	Lipid lowering treatment, n (%)		194 (22.6)
	Current smoking, n (%)		36 (4.2)
	Diabetes mellitus, n (%)		49 (5.7)
	Hypertension, n (%)		405 (47)
	SBP^a^ (mm Hg), mean (SD)		119 (14.1)
	DBP^b^ (mm Hg), mean (SD)		76 (8.5)
	Mean arterial pressure (mm Hg), mean (SD)		92 (10.8)
	**Arterial tonometry measures**		
		Carotid-femoral pulse wave velocity (m/second), mean (SD)	7.79 (1.79)
		Forward pressure wave amplitude (mm Hg), mean (SD)	47.6 (12.1)
		Reflection coefficient, mean (SD)	0.39 (0.07)
**Digital home BP^c^ during eFHS^d^ follow-up**			
	Follow-up weeks, median (IQR)		47 (21-52)
	Number of BP readings, median (IQR)		23 (10-47)
	Average SBP (mm Hg), mean (SD)		122 (12.3)
	Average DBP (mm Hg), mean (SD)		76 (8.2)
	ARV^e^ of SBP (mm Hg), mean (SD)		8.61 (3.34)
	ARV of DBP (mm Hg), mean (SD)		5.50 (2.22)

^a^SBP: systolic blood pressure.

^b^DBP: diastolic blood pressure.

^c^BP: blood pressure.

^d^eFHS: electronic Framingham Heart Study.

^e^ARV: average real variability.

### Association Between Arterial Stiffness Traits and Home BP Variability

**Figure 1:**
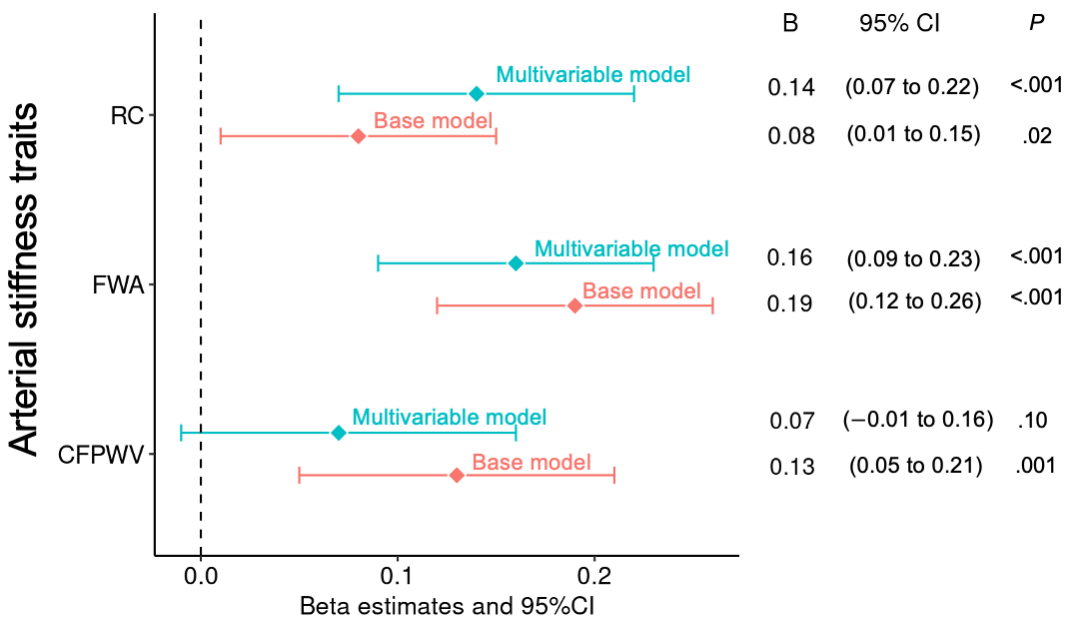
Association of arterial stiffness traits with ARV of home SBP in the base model and multivariable model. Covariates in base models include sex, age, and age squared. Covariates in the multivariable models include sex, age, age squared, BMI, height, heart rate, total cholesterol, high-density lipoprotein cholesterol, triglycerides, lipid-lowering treatment, fasting glucose, diabetes, current smoking, and antihypertensive medication. ARV: average real variability; CFPWV: carotid-femoral pulse wave velocity; FWA: forward pressure wave amplitude; RC: reflection coefficient; SBP: systolic blood pressure.

We observed that higher CFPWV, FWA, and RC were associated with higher ARV of week-to-week home SBP adjusting for sex, age, and age-squared in the base models (Figure 1). For example, we observed that 1 SD increments in FWA were associated with 0.19 SD increments in the ARV of home SBP. The association of FWA with ARV of home SBP was attenuated but persisted in the multivariable model that adjusted for additional covariates, albeit with a 16% reduction in the magnitude of association. The association between RC and ARV of home SBP was strengthened after including additional covariates in the multivariable model; however, the association between CFPWV and ARV of home SBP was attenuated and became nonsignificant. After further adjusting for MAP, the associations of FWA and RC with ARV of SBP were robust (attenuation: 44% and 43%, respectively; Multimedia Appendix 4).

**Figure 2:**
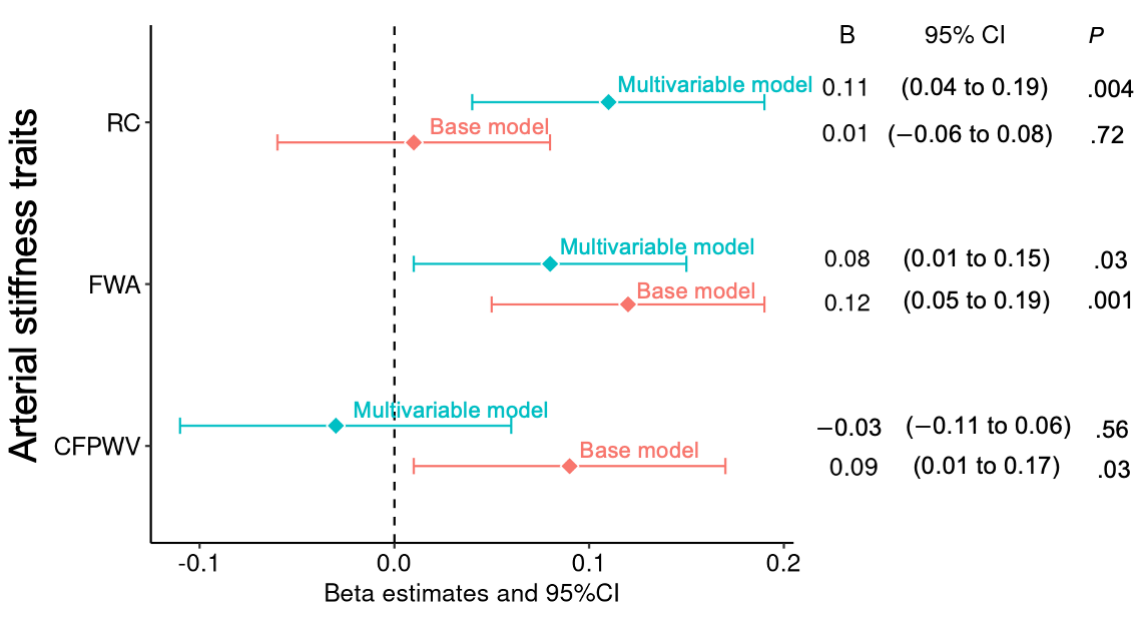
Association of arterial stiffness traits with ARV of home DBP in the base model and multivariable model. Covariates in base models include sex, age, and age squared. Covariates in the multivariable models include sex, age, age squared, BMI, height, heart rate, total cholesterol, high-density lipoprotein cholesterol, triglycerides, lipid-lowering treatment, fasting glucose, diabetes, current smoking, and antihypertensive medication. ARV: average real variability; CFPWV: carotid-femoral pulse wave velocity; DBP: diastolic blood pressure; FWA: forward pressure wave amplitude; RC: reflection coefficient.

Next, we performed association analyses between arterial stiffness traits and ARV of week-to-week home DBP (Figure 2). Higher CFPWV and FWA were associated with higher ARV of home DBP in the base models. However, we found no evidence of an association of RC with ARV of home DBP in the base models. The association of FWA with ARV of DBP persisted after adjustment for additional covariates (attenuation: 33%). Higher RC was associated with higher ARV of home DBP with a larger effect estimate, and CFPWV was no longer associated with ARV of home DBP in the multivariable model. When further adjusting for MAP, the directionality of the association between CFPWV and ARV of home DBP was reversed, resulting in higher CFPWV associated with lower ARV of DBP. Neither FWA nor RC was associated with ARV of DBP in the model that further considered MAP (Multimedia Appendix 5).

## Discussion

We investigated the association of arterial stiffness measures with mid- to long-term BPV defined as ARV of week-to-week home SBP and DBP collected using a digital BP cuff for up to 1-year follow-up among middle-aged to older community-dwelling adults with and without hypertension. Higher FWA and RC were associated with higher mid- to long-term ARV of home SBP after adjustment for important covariates (eg, antihypertensive use), while CFPWV was not associated with ARV of home SBP. Similarly, both FWA and RC exhibited positive associations with ARV of DBP in the multivariable models, while there was no evidence of an association of CFPWV with ARV of DBP. After further adjusting for MAP in the secondary analysis, the associations of FWA and RC with ARV of SBP were weakened but persisted compared with multivariable models without accounting for MAP, and we also observed an association between higher CFPWV and lower mid- to long-term ARV of DBP. This negative association relates to the opposing effects of MAP and CFPWV on DBP. An increase in MAP tends to increase DBP, while the MAP-related increase in CFPWV tends to increase PP and therefore decrease DBP [29]. As a result, when the aorta is compliant, changes in MAP will be directly reflected in commensurate changes in DBP. However, as the aorta stiffens, the effects of changes in MAP on DBP will be opposed by changes in PP, resulting in reduced DBP variability. Our findings suggest that measures of higher aortic stiffness and wave reflection were associated with week-to-week variation of BP in a home-based setting.

We used ARV due to its advantages over conventional indices like SD and CV. In comparison, ARV is an average of the absolute differences between consecutive BP measurements. It is more sensitive to the individual BP measurement sequence and may be a better index to represent short-term, reading-to-reading changes. For instance, a steady change (eg, 140, 130, 120, and 110) versus a more chaotic change (eg, 140, 120, 130, and 110) in BP will have the same mean, SD, and CV but a different ARV. Therefore, the ARV considers the order of the measurements and differences in consecutive BP measurements and therefore may be able to better reflect differences between steady change versus more dynamic change.

Central artery stiffness contributes to the pathogenesis of hypertension and the risk for target organ damage in the heart, kidneys, and brain. In contrast, while wave reflection can add to the load on the heart, it may be protective in the periphery by limiting the potentially harmful pulsatile energy transmitted to target organs [14,30]. Among Japanese adults with at least 1 risk factor for cardiovascular disease (CVD), day-to-day home BPV was associated with CVD events in adults with higher baseline arterial stiffness suggesting that arterial stiffness contributes to the association between home BPV and CVD risk [13] observed in a number of studies [6,8,31]. In the population-based Maastricht Study focusing on type 2 diabetes, 7-day systolic BPV was associated with aortic stiffness [32]. Similarly, a study of middle-aged Korean adults with high normal BP or hypertension, identified a significant relationship between home SBP variability and arterial stiffness. In that study, home BP measurements also occurred over 7 consecutive days. Our study extends these findings to home BP measurements taken week-to-week over a longer time horizon of up to 1 year and observed an association of arterial stiffness measures with mid- to long-term ARV of home BP.

The underlying pathophysiology of systolic versus diastolic BPV has been posited to be different [33]. The main distinction between SBP and DBP variability lies in the differing effects of PP variability and MAP variability. PP variability and MAP variability have additive effects on SBP and offsetting effects on DBP. The ARV of SBP correlates with arterial stiffness as observed in our study while ARV of DBP may be more related to endothelial dysfunction and impaired autonomic function [34].

Out-of-office home BP self-monitoring is a strategy that can be achieved in the community and in low-resource areas to improve hypertension awareness, treatment, and control and is supported by data from the International Databases on Ambulatory and Home Blood Pressure in Relation to Cardiovascular Outcomes [35] and endorsed by the American Heart Association and American College of Cardiology [1]. In addition, the ability to measure BP at home offers the patient the convenience of avoiding some office-based visits, empowers the patient to take multiple measurements over a longer period of time, and can improve engagement with BP management resulting in lower BP [36,37]. Using data from the National Health and Nutrition Examination Survey, less than 50% of people with known hypertension engaged in home BP monitoring at least monthly, leaving more work to be done [38]. With the rising use of mobile phones in the United States across diverse populations (White, Black, Hispanic, urban, and rural) [39], the extension of home BP measurements to digital measurements as in our study may permit the transmission of BP measurements to the health care team, development of educational visualization tools to enhance understanding of the BP measurement, and the use of nudges to encourage an individual to take BP measurements [40].

Our study had several strengths including the well-characterized community-based sample, the arterial stiffness measures obtained with a standardized protocol, and the tracking of BPs in the home setting using a digital device over up to 1 year. Higher SBP variability (visit-to-visit) is associated with adverse CVD outcomes in adults with optimal BP levels irrespective of underlying hypertension [12]. Therefore, it is critical to include individuals across the BP spectrum in studies that investigate BPV and other risk factors for CVD. Our study included individuals across a broad spectrum of BP levels, including those with and without underlying hypertension. This comprehensive inclusion enabled us to contribute valuable insights into understanding the potential biological mechanism that leads to increased risk of CVD with elevated BPV.

Our study also had some limitations that merit comment. First, more than half of the eFHS participants were excluded from our final sample, which led to differences in participant demographics between the study sample and the eFHS participants ineligible for our final analysis. The exclusion process potentially introduced a selection bias and limited the generalization of our findings to individuals of more diverse backgrounds. However, it is important to note that these 2 groups did not show significant differences in the prevalence of hypertension, reported use of antihypertensive medications, or measures of arterial stiffness, except for RC. This similarity may help mitigate the impact of selection bias to some extent. Second, the study is observational and cannot infer a causal relationship. Third, home BP measurements were taken by participants once per week at about the same time of the day each week. This schedule limited our ability to collect more frequent BP data, consequently restricting our ability to investigate BPV within a day or across different days within the same week. Advances in technology include wearables that measure BP at the wrist, which can provide more frequent data and a more convenient approach to collecting BP measurements than our method. However, more data are needed to determine the accuracy and usefulness of these devices. In addition, we did not correct for multiple statistical tests, which may lead to an inflated type I error. However, traditional correction techniques such as the Bonferroni correction would be overly conservative in our case due to correlated traits. Notably, upon applying multiple testing corrections, the results in our primary analysis remained largely unaffected, except for the association between ARV of home DBP and FWA, which subsequently lost its statistical significance.

In conclusion, in our middle-aged to older adult community-based sample, higher FWA and RC were associated with higher mid- to long-term ARV of home SBP and ARV of home DBP. Our findings suggest that higher aortic stiffness, as assessed by FWA, and greater relative wave reflection, as assessed by the global RC, may increase the week-to-week variation of home-based BP. The association of arterial stiffness with ARV of home BP may be a biological mechanism contributing to the increased risk of CVD associated with home BPV. Future work is needed to determine the potential beneficial effects on CVD outcomes of targeted attempts to reduce home BPV. The importance of defining the contribution to cardiovascular risk and outcomes of not only magnitude and duration but also variability of risk factors, including BP, is being recognized and requires additional work.

## Appendix

Multimedia Appendix 1 The timeline of data collection for arterial tonometry measures, as well as weekly home BP readings.

Multimedia Appendix 2 Flowchart of sample selection.

Multimedia Appendix 3 Comparison between three groups of samples.

Multimedia Appendix 4 Association of arterial stiffness traits with ARV of home SBP in the base model, multivariable model, and multivariable model that adjusted for MAP. Covariates in base models include sex, age and age squared. Covariates in the multivariable models include sex, age, age squared, BMI, height, heart rate, total cholesterol, high-density lipoprotein cholesterol, triglycerides, lipid-lowering treatment, fasting glucose, diabetes, current smoking, and anti-hypertensive medication. The multivariable model with MAP was further adjusted for MAP in addition to the variables in the multivariable model. ARV: average real variability; MAP: mean arterial pressure; RC: reflection coefficient; CFPWV: carotid femoral pulse wave velocity; FWA: forward pressure wave amplitude; SBP: systolic blood pressure.

Multimedia Appendix 5 Association of arterial stiffness traits with ARV of home DBP in the base model, multivariable model, and multivariable model that adjusted for MAP. Covariates in base models include sex, age and age squared. Covariates in the multivariable models include sex, age, age squared, BMI, height, heart rate, total cholesterol, high-density lipoprotein cholesterol, triglycerides, lipid-lowering treatment, fasting glucose, diabetes, current smoking, and anti-hypertensive medication. The multivariable model with MAP was further adjusted for MAP in addition to the variables in the multivariable model. ARV: average real variability; MAP: mean arterial pressure; RC: reflection coefficient; CFPWV: carotid femoral pulse wave velocity; FWA: forward pressure wave amplitude; DBP: diastolic blood pressure.

## Abbreviations

ARV: average real variability
BP: blood pressure
BPV: blood pressure variability
CFPWV: carotid-femoral pulse wave velocity
CV: coefficient of variation
CVD: cardiovascular disease
DBP: diastolic blood pressure
eFHS: electronic Framingham Heart Study
FHS: Framingham Heart Study
FWA: forward pressure wave amplitude
Gen 3: third generation
MAP: mean arterial pressure
PP: pulse pressure
RC: reflection coefficient
SBP: systolic blood pressure

## Data Availability

The data sets generated and analyzed during this study are available from the corresponding author on reasonable request and will be available at public repositories (DbGAP and BioLINNC).
