# Supplementary figures and images for: Association of Arterial Stiffness With Mid- to Long-Term Home Blood Pressure Variability in the Electronic Framingham Heart Study: Cohort Study
Source: JMIR Cardio. 2024 Apr 8;8:e54801. doi: 10.2196/54801 (PMC11036191; doi:10.2196/54801)

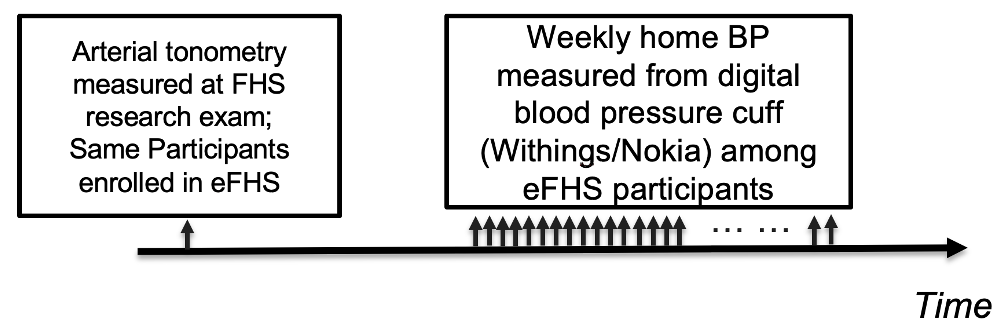

Supplement: Multimedia Appendix 1 [file cardio_v8i1e54801_app1.png]

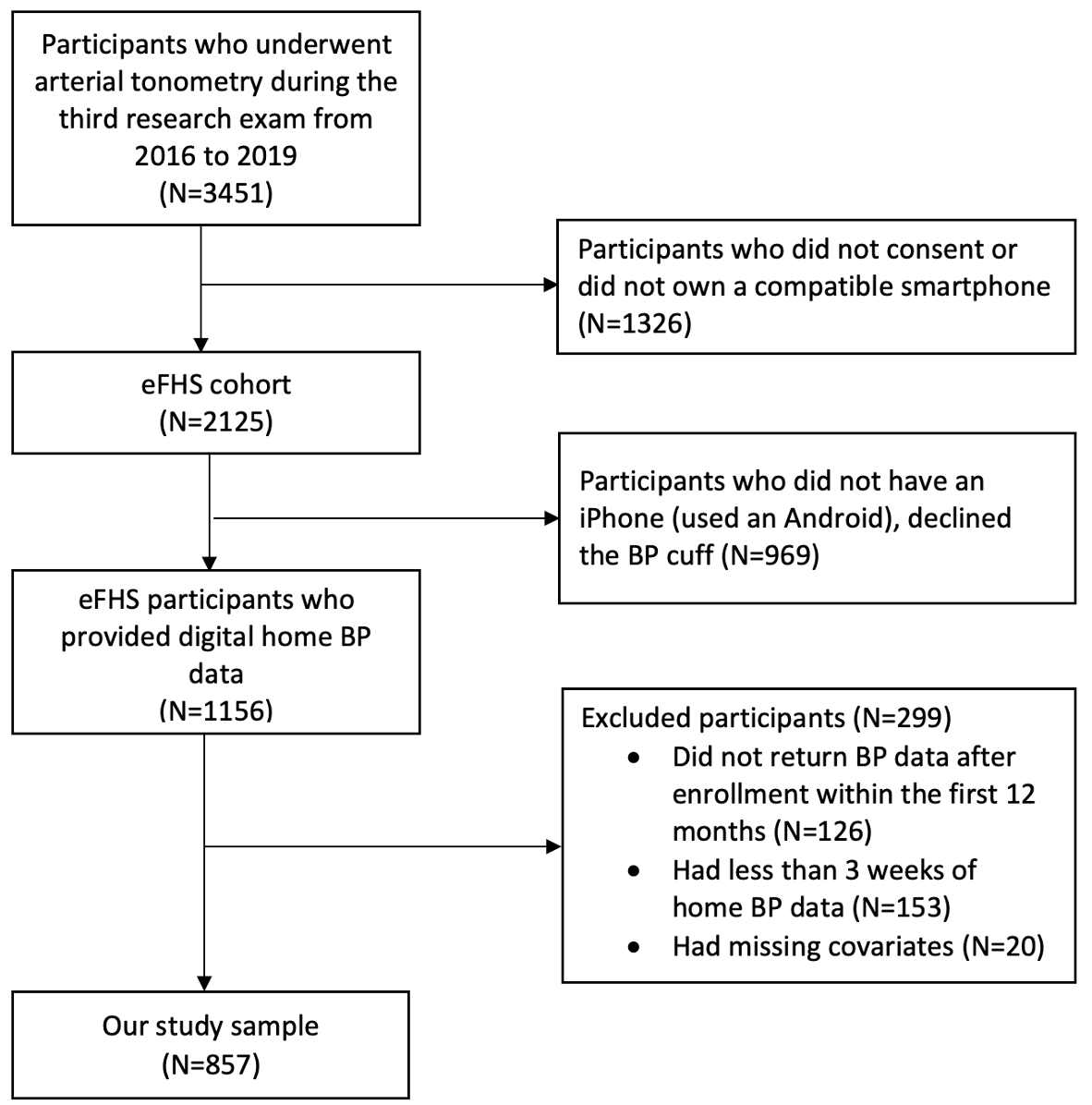

Supplement: Multimedia Appendix 2 [file cardio_v8i1e54801_app2.png]

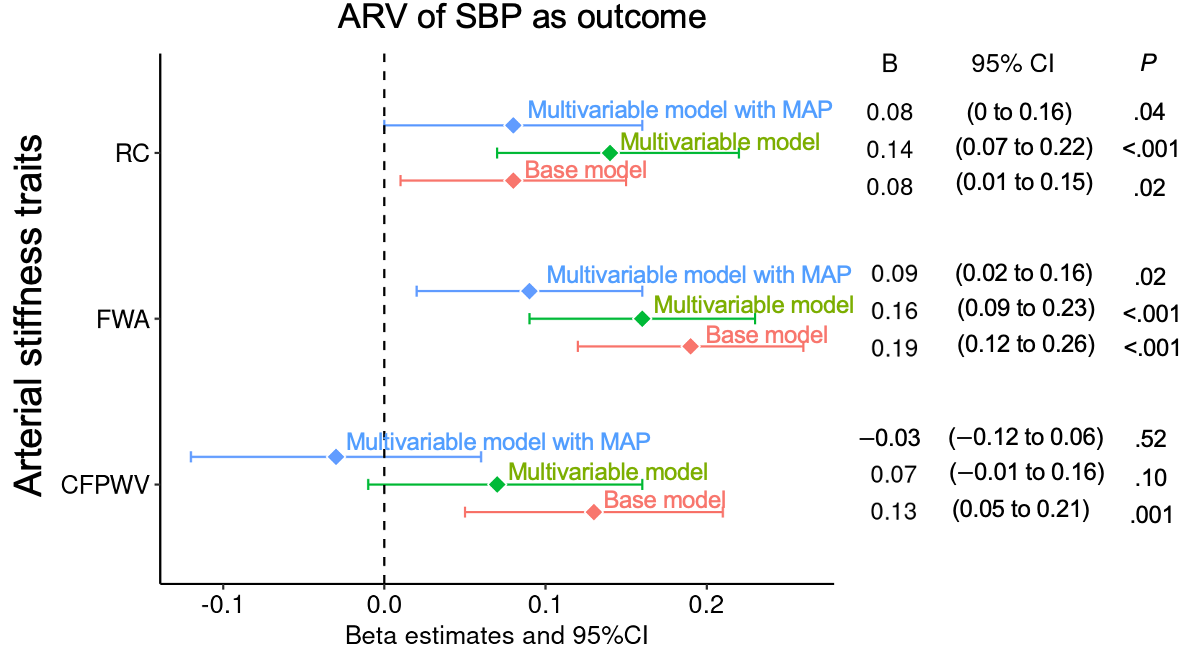

Supplement: Multimedia Appendix 4 [file cardio_v8i1e54801_app4.png]

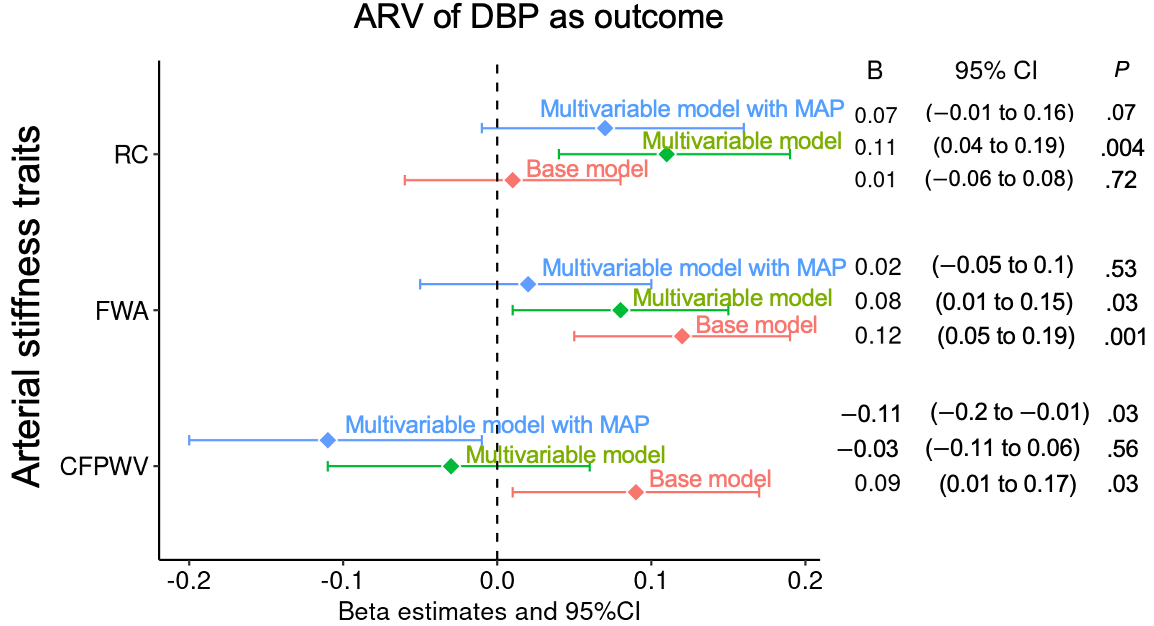

Supplement: Multimedia Appendix 5 [file cardio_v8i1e54801_app5.png]
