# Supplementary material for: Association of Arterial Stiffness With Mid- to Long-Term Home Blood Pressure Variability in the Electronic Framingham Heart Study: Cohort Study
Source: JMIR Cardio. 2024 Apr 8;8:e54801. doi: 10.2196/54801 (PMC11036191; doi:10.2196/54801)
Supplement: Multimedia Appendix 3 [file cardio_v8i1e54801_app3.docx]

**Multimedia Appendix 3.** Comparison between three groups of samples.

| **Group** | **1** | **2** | **3** |  |  |
| --- | --- | --- | --- | --- | --- |
| **Variables at exam 3** | **FHS participants who did not enroll in eFHS** | **eFHS participants ineligible for final analysis** | **eFHS participants in final sample** | ***P* value: group 1 vs 3** | ***P* value: group 2 vs 3** |
| Sample Size | 1326 | 1268 | 857 |  |  |
| Age (years), mean (SD) | 57 (9.53) | 52.4 (8.8) | 53.5 (8.56) | <.001 | .003 |
| Sex female, n (%) | 662 (50) | 686 (54.1) | 508 (59) | <.001 | .02 |
| BMI (kg/m^2^), mean (SD) | 28.9 (6.0) | 28.8 (6.26) | 27.6 (4.79) | <.001 | <.001 |
| Carotid femoral pulse wave velocity (CFPWV) (m/s), mean (SD) | 8.55 (2.54) | 7.75 (1.73) | 7.79 (1.79) | <.001 | .61 |
| Forward pressure wave amplitude (FWA) (mmHg), mean (SD) | 50.3 (13.1) | 47.1 (11.5) | 47.6 (12.1) | .001 | .33 |
| Reflection coefficient (RC), mean (SD) | 0.38 (0.07) | 0.38 (0.07) | 0.39 (0.07) | <.001 | <.001 |
| Heart Rate (BPM), mean (SD) | 60.1 (10.1) | 59.1 (9.54) | 58 (9.08) | <.001 | .007 |
| Systolic BP (mmHg), mean (SD) | 121 (14.8) | 118 (13.4) | 119 (14.1) | <.001 | .15 |
| Diastolic BP (mmHg), mean (SD) | 75.8 (9.0) | 75.6 (8.33) | 76.1 (8.49) | .43 | .22 |
| Mean arterial pressure (mmHg), mean (SD) | 93 (11.0) | 91.9 (10.9) | 91.9 (10.8) | .03 | .96 |
| Current smoking, n (%) | 103 (7.8) | 87 (6.9) | 36 (4.2) | .001 | .01 |
| Diabetes mellitus, n (%) | 168 (12.7) | 88 (6.9) | 49 (5.7) | <.001 | .28 |
| Hypertension, n (%) | 759 (57.2) | 570 (45) | 405 (47.3) | <.001 | .32 |
| Total cholesterol (TC) (mg/dL), mean (SD) | 187 (37.8) | 191 (35.5) | 190 (35.8) | .13 | .40 |
| High-density lipoprotein cholesterol (HDL) (mg/dL), mean (SD) | 58.1 (19.4) | 59.2 (18.9) | 62.0 (19.9) | <.001 | .001 |
| Triglycerides (mg/dL), median (25^th^, 75^th^ percentile) | 97 (31, 983) | 93 (21, 1360) | 87 (26, 569) | <.001 | <.001 |
| Fasting blood glucose (mg/dL), mean (SD) | 103 (25.9) | 99.3 (20.2) | 98 (17.9) | <.001 | .09 |
| Antihypertensive use, n (%) | 397 (29.9) | 253 (20) | 189 (22.1) | <.001 | .26 |
| Lipid lowering treatment, n (%) | 369 (27.8) | 250 (19.7) | 194 (22.6) | .008 | .12 |
